# Hydroxyurea synergizes with valproic acid in wild-type p53 acute myeloid leukaemia

**DOI:** 10.18632/oncotarget.6991

**Published:** 2016-01-23

**Authors:** Calum Leitch, Tereza Osdal, Vibeke Andresen, Maren Molland, Silje Kristiansen, Xuan Nhi Nguyen, Øystein Bruserud, Bjørn Tore Gjertsen, Emmet McCormack

**Affiliations:** ^1^ Department of Clinical Science, University of Bergen, Bergen, N-5021 Norway; ^2^ Centre of Cancer Biomarkers, Department of Clinical Science, University of Bergen, Bergen, N-5021 Norway; ^3^ Department of Internal Medicine, Hematology Section, Haukeland University Hospital, Bergen, N-5021 Norway

**Keywords:** valproic acid, hydroxyurea, AML, DNA damage, p53

## Abstract

Palliative care in acute myeloid leukaemia (AML) is inadequate. For elderly patients, unfit for intensive chemotherapy, median survival is 2–3 months. As such, there is urgent demand for low-toxic palliative alternatives. We have repositioned two commonly administered anti-leukaemia drugs, valproic acid (VPA) and hydroxyurea (HU), as a combination therapy in AML.

The anti-leukemic effect of VPA and HU was assessed in multiple AML cell lines confirming the superior anti-leukemic effect of combination therapy. Mechanistic studies revealed that VPA amplified the ability of HU to slow S-phase progression and this correlated with significantly increased DNA damage. VPA was also shown to reduce expression of the DNA repair protein, Rad51. Interestingly, the tumour suppressor protein p53 was revealed to mitigate cell cycle recovery following combination induced arrest. The efficacy of combination therapy was validated *in vivo*. Combination treatment increased survival in OCI-AML3 and patient-derived xenograft mouse models of AML. Therapy response was confirmed by optical imaging with multiplexed near-infrared labelled antibodies.

The combination of HU and VPA indicates significant potential in preclinical models of AML. Both compounds are widely available and well tolerated. We believe that repositioning this combination could significantly enhance the palliative care of patients unsuited to intensive chemotherapy.

## INTRODUCTION

Palliative care in acute myeloid leukaemia (AML) is unsatisfactory. For elderly patients, who do not tolerate intensive chemotherapy or bone marrow transplantation, median survival is 2–3 months [1, 2]. AML is a genetically heterogeneous disease and numerous classes of anticancer agents have been trialled with varying success. Histone deacetylase inhibitors (HDACi) have indicated significant potential, whilst DNA targeting compounds remain a stalwart of clinical practice [3]. Emerging studies continue to reveal novel molecular mechanisms underpinning these agents. Such molecular insights also provide a unique opportunity to reassess existing therapeutics.

Valproic acid (VPA) is a short chain fatty acid used clinically as an anticonvulsant for more than 30 years. In 2001 the compound was rediscovered for its anticancer activity as an HDAC inhibitor, (HDACi) targeting class 1 and 2 HDAC enzymes [4]. A multitude of preclinical studies have combined VPA with genotoxic and non-genotoxic therapies reporting substantially increased efficacy [5–8]. More recently it has been illustrated that this increased efficacy may be accountable to VPA's capacity to modulate DNA damage repair proteins. Specifically VPA has been shown to target homologous recombination proteins, negatively regulating expression and localization of Rad51, Chk1, BRCA1 and BRCA2. [9] In the last decade various clinical trials have exhibited VPA's capacity to incite clinical response in primary and secondary AML patients [10–13]. However, the use of VPA in AML is predicated on its HDACi activity and consequent induction of differentiation and apoptosis. Limited clinical emphasis has been placed on its ability to modulate DNA repair.

Hydroxyurea (HU) is an antimetabolite that targets cancer cells through stalling and subsequent collapse of S-phase replication forks [14]. HU inhibits ribonucelotide reductase thereby depleting the cellular pool of deoxynucleotides and incurring reversible DNA damage [15]. S-phase specific genomic assaults then depend on homologous recombination proteins, including Rad51, for repair [16]. The clinical importance of HU in myeloid neoplasms cannot be understated. Hydroxyurea is routinely administered to achieve leukocytoreduction in AML, chronic myeloid leukaemia, hyperleukocytosis and leukostasis [17, 18]. The safety of the compound is further exemplified by its on-going use and reccomendation for the treatment of young patients suffering from sickle cell anaemia [19]. In AML the compound may be prescribed prior to induction chemotherapy or experimentally in combination with other moderate or low toxic therapies, particularly in palliative care [20, 21].

Various preclinical studies have examined the synergistic effects of combining genotoxic compounds and HDACi [22–25]. However, the mechanisms described to account for the observed synergism remain inconclusive and may differ substantially depending on the models explored. Based on our preclinical and clinical experience with VPA we are appreciating its relevance in palliative care of AML [5, 26, 27]. Furthermore non-systematic testing of VPA and HU in single patients suggests the combination is well tolerated in elderly patients [21]. These factors encouraged us to reassess HU and VPA as a combination in preclinical models of AML. Here we provide evidence that HU and VPA combine effectively in AML through cooperative modulation of the cell cycle and DNA repair proteins. Additionally we identify intact tumour suppressor protein p53 as a likely predictor of therapeutic response to the combination. *In vivo* imaging and survival analysis in orthotopic mouse models, including a patient-derived xenograft model, confirmed that this combination treatment improves survival. The established tolerance and low toxicity of these compounds additionally highlights their potential in the palliative care of elderly AML patients.

## RESULTS

### HU and VPA cooperatively induce cell death in p53 wild-type leukaemia cell lines

**Figure 1:**
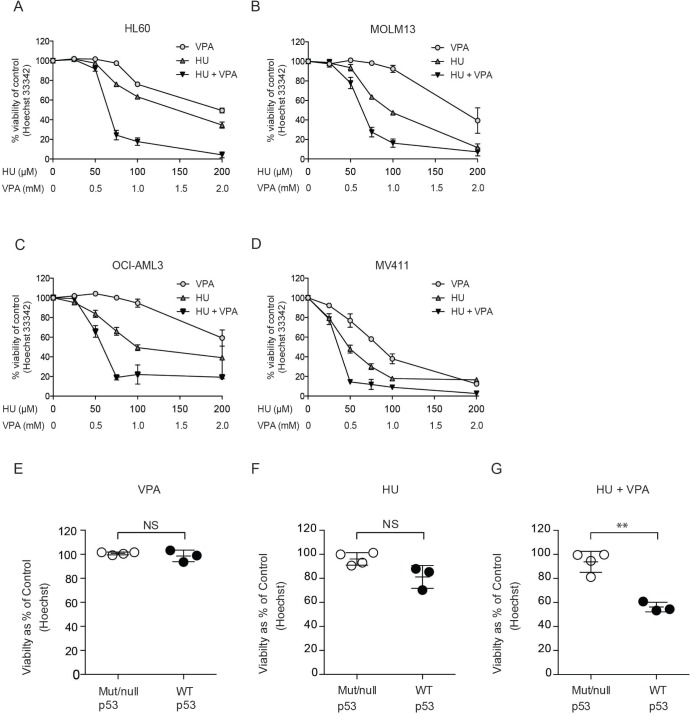
Assessment of cell death induction and the enhanced potential of combining HU and VPA in AML cell lines (**A-D**) Hoechst nuclear staining assay performed to generate dose response curves for cell death induction. HL-60, MOLM-13, OCI-AML3 and MV4-11 cells were treated with HU (25–200 μM) and VPA (0.25–2 mM) alone or in combination at a fixed ratio (1:10) for 72 hrs. *N* = 3. (**E-G**) Hoechst nuclear staining assay is performed to determine the % of viable cells in MOLM-13, OCI-AML3, MV4-11 (p53 wild-type), KG1-A, THP-1, K562 (p53 mutated) and HL-60 cell lines. All cell lines were treated with HU (60 μM), VPA (0.6 mM) and the combination. Results are pooled and presented as mutated/null p53 (Mut/null p53) vs. wild-type p53 cell lines (WT p53). A significant increase in sensitivity was observed in WT p53 cell lines under combination treatment (**P < 0.01) *N* = 3.

**Figure 2:**
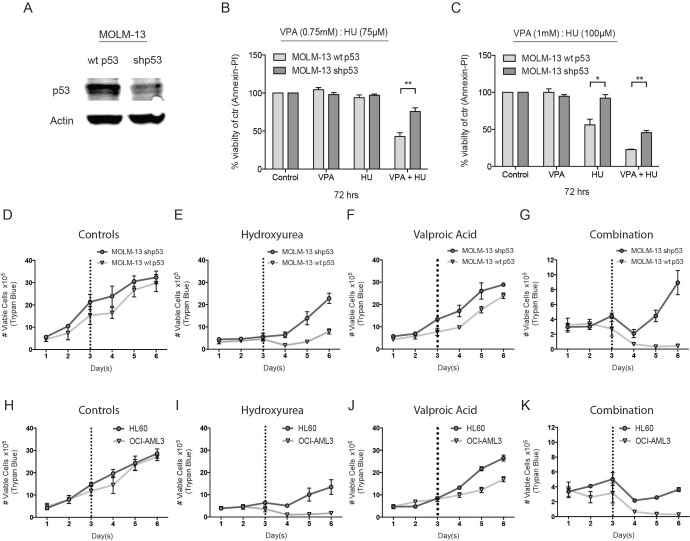
Investigating the role of p53 in HU and VPA combination therapy (**A**) Lysate was produced from untreated MOLM-13 wt and shp53 cells. Immunoblotting was performed with antibodies targeting p53 and actin. *N* = 3. (**B**) + (**C**) MOLM-13 wt and shp53 cells were treated with HU (75 μM and 100 μM), VPA (0.75 mM and 1 mM) and the combination of both for 72 hrs and apoptosis was determined by Annexin-PI to generate dose response curves. Induction of apoptosis was compared between each cell line at particular treatment conditions (*P < 0.05, **P < 0.01). *N* = 3. (**D–G**) MOLM-13 wt and shp53 cells and (**H-K**) HL-60 and OCI-AML3 cells were treated with HU (100 μM) and VPA (1 mM), the combination or seeded without treatment. Following 72 hrs cells were washed 2 × in sterile saline and reseeded in wells. The cells were followed for a further 3 days and the number of viable cells was determined at 24 hrs intervals throughout the entire experimental course. Cell counts were performed using the Countess™ Automated Cell Counter (Invitrogen). *N* = 3.

The cell death capacity of HU and VPA alone and in combination was assessed in four AML cell lines (MV4– 11, OCI-AML3, MOLM-13, and HL-60) using Hoechst 33342 nuclear staining. Cells were treated at a fixed ratio alone or in combination for 72 hours with increasing doses of HU (25–200 μM) and VPA (0.25–2 mM) (Figure 1A–1D). Combination treatment consistently enhanced cell death induction as compared to the single agents in all cell lines. However, when comparing the cell viability at doses (HU 50 μM and VPA 0.5 mM) best reflecting patient serum concentrations [10, 21], the p53 null HL-60 cells were identified as the most resistant cell line (Figure 1A–1D). To examine whether p53 status can mediate therapy sensitivity at clinically relevant doses, 3 additional leukemic cell lines (KG1-A, THP-1 and K562) harbouring p53 mutations were assessed and compared to the cell lines previously described. All cell lines were exposed to HU 60 μM and VPA 0.6 mM for 72 hours to reflect clinically achievable concentrations [10]. Cell death in response to combination therapy was significantly increased in wild-type p53 cell lines compared to null or mutated p53 cell lines. Comparatively, single agent therapy failed to distinguish significantly between cell lines with varying p53 status (Figure 1E–1G). To further investigate the significance of p53 status in response to HU and VPA combination therapy, we employed MOLM-13 cells expressing shRNA targeting p53 gene expression. Western blotting confirmed reduced expression of the p53 protein in MOLM-13 shp53 cells when compared with MOLM- 13 wt p53 cells transduced with an untargeted empty vector (Figure 2A) The two cell lines were treated with HU (75 μM and 100 μM), VPA (0.75 mM and 1 mM) or the combinations. Cell death was determined by flow cytometry using Annexin-PI staining following 72 hrs treatment (Figure 2B–2C). At both concentration ratios, the combination therapy induced significantly more death in MOLM-13 wt p53 cells when compared with MOLM- 13 shp53 cells. It is a growing concern that chemotherapy may select for a minority of p53 mutant clones in AML patients [28]. This may contribute significantly to the emergence of therapy resistant relapse disease. To investigate the enduring effect of the combination therapy, cells were exposed to HU (100 μM), VPA (1 mM) and the combination for 72 hrs. Cells were then washed twice and reseeded in drug free medium and maintained for a further 72 hrs. Viable cells were counted at 24 hr intervals throughout the course of the experiment (6 days). This recovery assay was performed in MOLM-13 shp53, MOLM-13 wt p53 (Figure 2D–2G), HL-60 (p53^null^) and OCI-AML3 (p53^wild-type^) cells (Figure 2H–2K). In all cell lines untreated control cells displayed typical growth curves over the 6 day period, whilst VPA exerted a mild slowing of division rate that was lost with removal of the treatment. HU exhibited a more profound arrest in cell division, particularly in cells with wild-type p53 status. However, again all cell lines were able to recover upon removal of the treatment. Uniquely, the combination therapy limited recovery to the HL-60 and MOLM-13 shp53 cell lines, with treatment resulting in a terminal arrest of MOLM-13 wtp53 and OCI-AML3 cells. The presence of substantial p53 expression therefore appears crucial to induction of a lasting anti-leukemic effect with this combination.

### HU and VPA cooperatively regulate cell cycle in OCI-AML3

**Figure 3:**
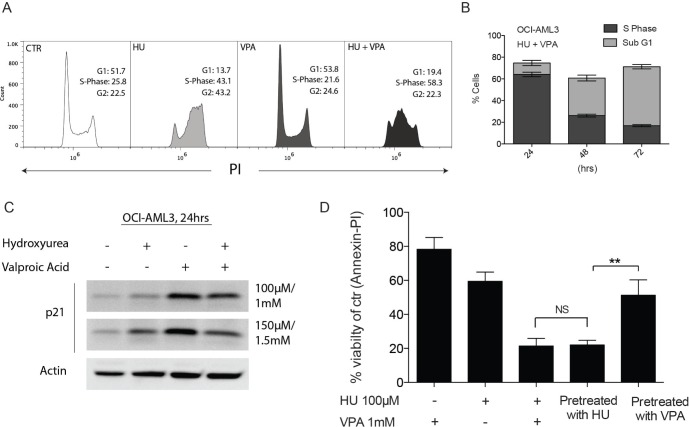
Complimentary regulation of cell cycle status following combination therapy in OCI-AML3 cells (**A**) OCI- AML3 cells were treated with HU (100 μM), VPA (1 mM) and the combination of both for 24 hrs before cells were stained with PI and cell cycle status analysed by flow cytometry. Analyses was performed in 3 independent experiments with Figure (**A**) providing representative plots. (**B**) Comparison of OCI-AML3 cell populations defined as S-phase or Sub G1 (necrotic) at 24 hrs, 48 hrs and 72 hrs combination treatment with HU (100 μM) and VPA (1 mM). *N* = 3. (**C**) OCI-AML3 cells were incubated for 24 hrs with doses of HU (100 μM and 150 μM) and VPA (1 mM and 1.5 mM). Immunoblotting was with antibodies towards p21 and actin. (**D**) OCI-AML3 cells were treated with HU (100 μM), VPA (1 mM) and the combination of both for 72 hrs. Combination experiments were performed where monotherapies where added for the first 24 hrs with the secondary treatment added for the final 48 hrs of the experiment (Pretreatment with HU/VPA). Apoptosis was determined by Annexin-PI. *N* = 3. The two alternate sequence studies proved to significantly affect the compounds capacity to induce apoptosis (**P < 0.01).

Given the apparent significance of the role of p53 in combination treatment response, OCI-AML3 (p53 wild- type) cells were selected for mechanism of action studies. Previous studies have determined that the combination of HU and VPA can cooperatively arrest cell cycle progression resulting in enhanced cell death [23]. We assessed the cell cycle status of OCI-AML3 cells following 24 hrs exposure to HU (100 μM) and VPA (1 mM), both alone and in combination (Figure 3A). HU slowed cell cycle progression with OCI-AML3 cells accumulating in S and G2/M phase. VPA's capacity to induce G1 arrest is previously described [29], however OCI-AML3 cells were only mildly arrested following VPA exposure for 24 hrs (Control 51.7% vs. VPA 53.8%). Combination therapy resulted in strong S-phase arrest where the presence of VPA appears to amplify HU's capacity to slow S-phase to G2 progression. Figure 3B illustrates that the percentage of cells located in S-phase at 24 hrs combination therapy is consistent with the fraction of cells determined to be necrotic (sub G1) following 72 hrs drug exposure. It is previously described that HU mitigates HDACi induction of the cyclin-dependent kinase inhibitor p21 protein enabling cooperative initiation of S-phase driven apoptosis in cancer cells [23]. This trend was observed in the OCI- AML3 cells (Figure 3C). The capacity of VPA to induce p21 and simultaneously arrest cells in G1 increases significantly over time. To assess whether VPA had the capacity to protect cells from HU driven apoptosis, cells were pretreated with HU (100 μM) or VPA (1 mM) for 24 hrs prior to addition of the second complementary compound for a further 48 hrs. Cell viability was assessed by Annexin-PI flow cytometry. Strikingly, when pretreated with VPA for 24 hrs cells were significantly more resistant as compared to HU pretreatment (Figure 3D). These results suggest that unmitigated entry to S-phase is necessary for synergistic induction of cell death. The indication that S-phase arrest is important to establish the synergistic effect of combination therapy is emphasized when cell cycle status of OCI-AML3 cells is followed over 72 hrs of combination treatment (Figure 3D).

### HU and VPA in combination target DNA damage repair proteins mitigating recovery from DNA double strand breaks

**Figure 4:**
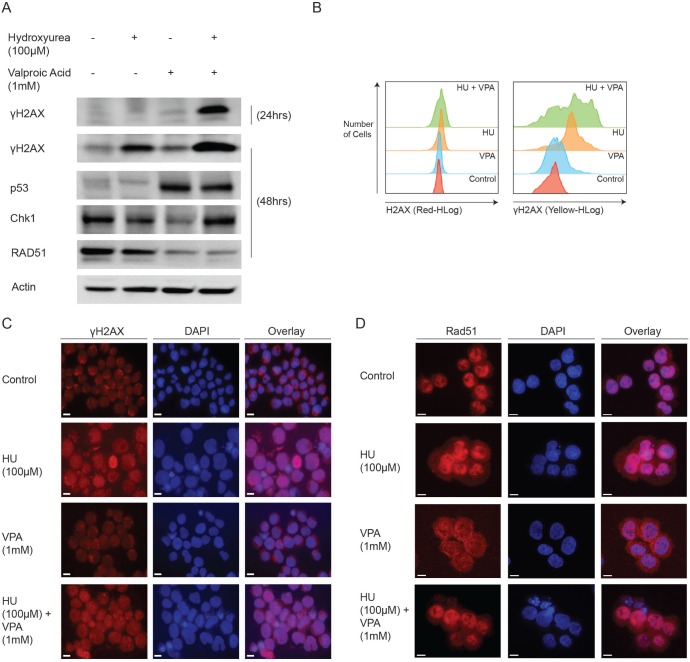
Mechanistic studies demonstrate the combinations capacity to regulate DNA damage repair proteins (**A**) OCI-AML3 cells were treated with HU (100 μM), VPA (1 mM) and the combination of both for 24 and 48 hrs. Immunoblotting was performed using antibodies towards γH2AX, p53, Chk1, Rad51 and actin. *N* = 3. (**B**) OCI-AML3 cells were treated with HU (100 μM), VPA (1 mM) and the combination of both for 24 hrs. Assesment of H2AX and γH2AX expression was performed using flow cytometry with the Muse™ H2A.X Activation Dual Detection Kit. *N* = 3. (**C** + **D**) OCI-AML3 cells were treated for 48 hrs with HU (100 μM), VPA (1 mM) or in combination, before cytospun, fixed and immunostained for Rad51. Representative image of three independent experiments is shown.

To determine whether S-phase arrest was associated with anticipated HU driven DNA double strand breaks (DSBs), we evaluated the expression of various DNA damage repair proteins following exposure to both compounds and in combination. Protein expression was evaluated at 24 or 48 hrs to ensure > 65% of cells were viable. Immunoblotting revealed a marked increase in the DNA DSB indicator protein γH2AX following combination treatment as compared to monotherapies at both 24 and 48 hrs (Figure 4A). Flow cytometry verified these results and demonstrated additionally that the increased γH2AX was due to the phosphorylated version of the H2AX protein (Figure 4B). Interestingly the tumour suppressor protein p53 was unresponsive to HU treatment alone, but as expected expression was significantly increased upon VPA treatment and this was sustained in combination therapy. Chk1 was decreased in VPA monotherapy [9, 30], however expression appeared unaffected in the combinatory treatment, likely due to the increased presence of DSBs. Crucially, the homologous recombination repair protein, Rad51 was reduced in the presence of VPA, both alone and in combination with HU. Furthermore, immunofluorescence staining revealed γH2AX foci formation in the nucleus of cells exposed to HU or combination (Figure 4C). Additional localisation studies verified the formation of the DNA damage repair foci by nuclear imaging of the MRN complex member, Nsb-1, in HU and combination treated cells (Supplementary Figure 1A). Immunofluorescence staining for Rad51 indicated the protein is reduced and restricted to the cytoplasm in VPA treated cells (Figure 4D). The cytoplasmic restriction of Rad51 following VPA treatment was marked, while Rad51 expression in combination treated cells was diffuse and unspecific. Together these results suggest that the capacity of HU to induce DNA DSBs is vastly enhanced by the presence of VPA, likely due to aberrant expression and localization of Rad51.

### Cell death studies confirm synergistic capacity of HU and VPA in OCI-AML3 and primary AML blasts

**Figure 5:**
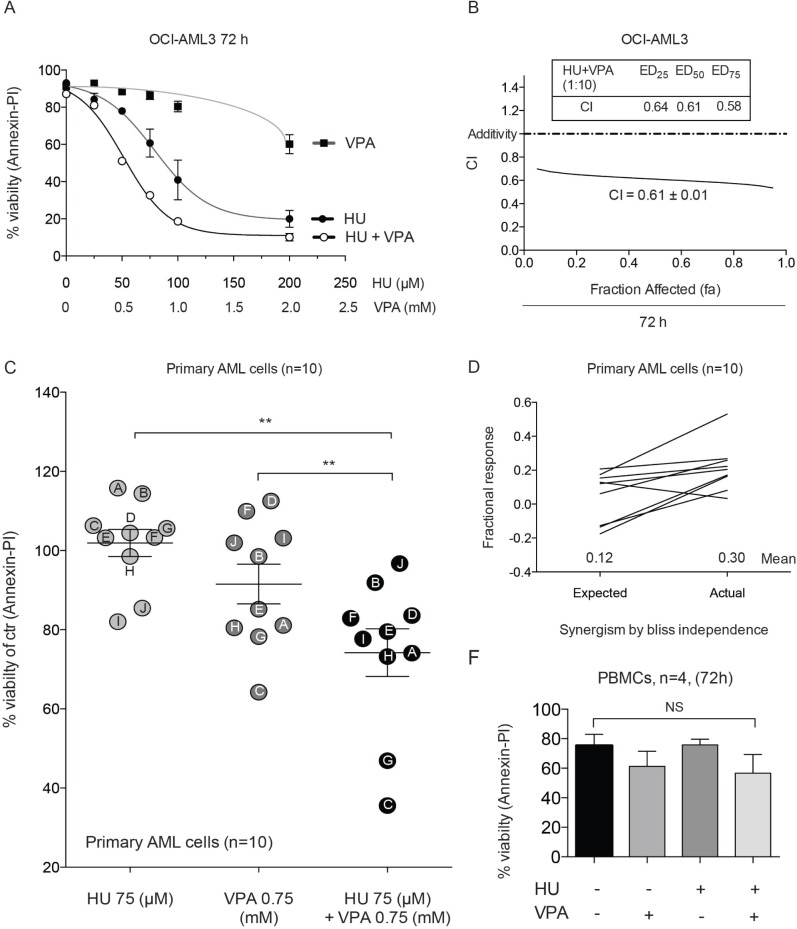
Annexin-PI studies demonstrate synergy in OCI-AML3, primary AML blasts and the non-toxic effect of the combination in PBMCs (**A**) OCI-AML3 cells were treated with increasing doses of HU (0–200 μM) and VPA (0–2 mM) alone or in combination at a fixed ratio (1:10) for 72 hrs and apoptosis was determined by Annexin-PI to generate dose response curves. *N* = 3. (**B**) The data generated in figure (A) enabled CI vales to be plotted at fa (0–1.0). (**C**) Differences in means of viability between HU (75 μM), VPA (0.75 mM) and combination of both for 24 hrs for the pooled patient data analyzed by Annexin-PI. Results are given as means ± s.e. of mean (**P < 0.01, *n* = 10). (**D**) Bliss Independence analysis of expected and actual response for the combinational therapy of HU (75 μM) and VPA (0.75 mM) for each of the individual AML patient samples analyzed by Annexin-PI. (**E**) Peripheral blood mononucleocytes (PBMCs) obtained from four healthy donors were treated with HU (100 μM), VPA (1 mM) and the combination of both for 72 hrs and apoptosis was determined by Annexin-PI. Reduction of viability by the combinational treatment was compared with untreated control cells to reveal a non-significant (NS).

Prior to performing preclinical studies *in vivo*, we wished to corroborate the capacity of HU and VPA to synergistically induce cell death in OCI-AML3 cells and primary AML blasts whilst remaining non-toxic in healthy peripheral blood mononuclear cells. Flow cytometry assessment of Annexin-PI expression was performed to determine induction of cell death. Dose response curves and combination index analysis in OCI- AML3 cells supported the Hoechst 33342 data presented in Figure 1C (Figure 5A and 5B). Primary AML cells from 10 randomly selected AML patients exhibited a range of sensitivity to HU (75 μM) and VPA (0.75 mM) alone or in combination when treated for 24 hrs (Figure 5C, Table 1). Combining the results from all ten patients demonstrated a significant reduction in mean viability for the HU and VPA combination treatment against either compound alone (Figure 5C). Furthermore, synergism, as calculated by Bliss Independence, was obtained in 9 of the 10 patients samples assessed (Figure 5D). The viability of PBMCs from 4 healthy donors exposed to the HU or VPA alone or in combination for 72 hrs was determined by Annexin- PI staining to reveal no significant increase in cell death (Figure 5E).

**Table 1:** Clinical and biological characteristics of the 11 AML patients included in the study

Gender	Age	Previous	FAB	CD34	Cytogenetics	FLT3	NPM-1	P53	Synergy Ranking	Patient ID
M	32	*De novo*	M3	Positive	t(15;17)	ITD	wt	wt	1	G
F	87	*De novo*	M0	Positive	Del 5 (q13, q33)	Normal	nt	wt	2	D
M	78	*De novo*	M4	Negative	+8	Nt	nt	wt	3	C
F	29	*De novo*	M5	Positive	Normal	ITD+Asp835	wt	wt	4	F
M	76	MDS	nt	Positive	Normal	nt	nt	nt	5	B
M	82	*De novo*	nt	Positive	+8	wt	wt	wt	6	A
F	78	*De novo*	M1	Negative	Normal	ITD	Ins	wt	7	E
F	18	*De novo*	M4	Positive	Inv16	wt	wt	wt	8	I
M	64	*De novo*	M5	Negative	Normal	wt	Ins	nt	9	H
M	76	MDS	nt	Positive	Normal	nt	nt	wt	10	J
F	59	*De novo*	M4	Positive	Normal	ITD	Ins	wt	nt	PDX

**Figure 6:**
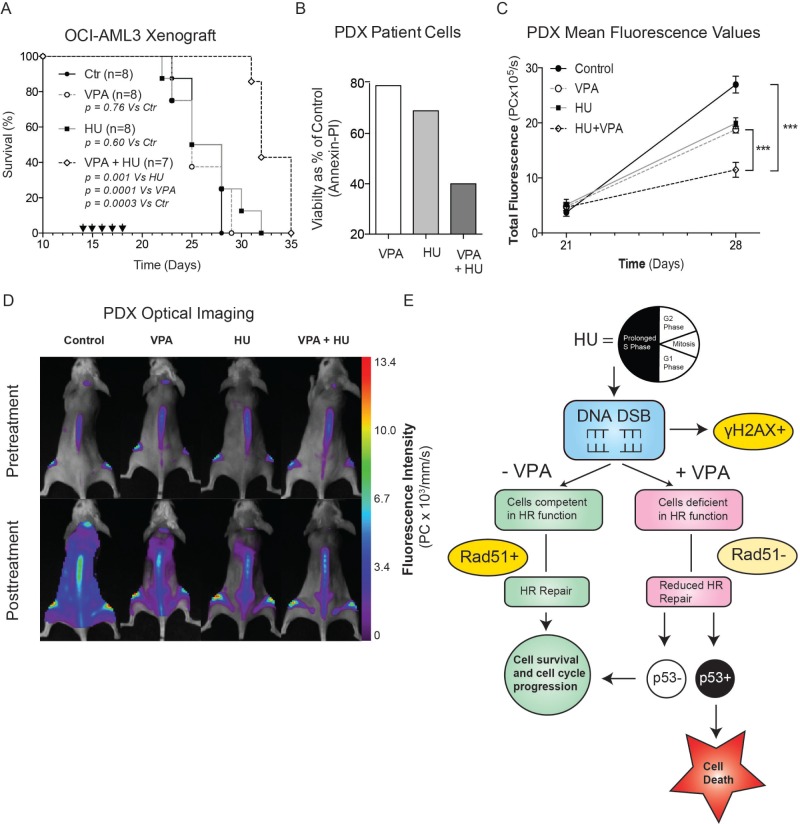
Combinational therapy of HU and VPA represses AML *in vivo* (**A**) Survival data presented in Kaplan-Meyer curve illustrating the efficacy of HU and VPA and increased survival of combination therapy (log-rank *P* = 0.0003 vs controls, *P* = 0.0014 vs HU, *P* = 0.0001 vs VPA) in the OCI-AML3 orthotopic model of AML. Arrows indicate days on which animals were dosed with both compounds. Control (*n* = 8), HU (*n* = 8), VPA (*n* = 8) and combination (*n* = 7). (**B**) Primary AML cells from the same generation used to generate the PDX model were thawed and treated with HU (75 μM), VPA (0,75 mM) and the combination of both for 24 h and apoptosis determined by Annexin-PI staining. (**C** + **D**) Imaging with multiplexed mAbs was performed before initiation of therapy (day 21) and 1 week later (day 28). Control (*n* = 8), HU (*n* = 8), VPA (*n* = 8) and combination (*n* = 6). Representative images of mice after treatment reveal a significant reduction in total fluorescence (photon count per second) of combination treated mice compared with vehicle controls and monotherapies. Combination compared with control (*p* < 0.0001), VPA (*p* = 0.0002) and HU (*p* = 0.0005). (**E**) Schematic overview of the combination mechanism of action and key molecular events determining cell death or survival in AML cells exposed to HU and VPA. HU, Hydroxyurea; VPA, Valproic Acid; DSB, Double strand breaks; HR, homologous recombination.

FAB, French-American-British; F, Female; FLT3, FMS-like tyrosine kinase-3; Ins, Insertion; ITD, Internal tandem duplicate; M, Male; MDS, Myelodysplastic syndrome; NPM1, Nucleophosmin-1; nt, Not tested; PDX, Refers to patient cells used to generate patient derived xenograft model implemented in Figures 6B and 6C; wt, wild type. Synergy ranking determined by the difference in actual vs. expected viability values using bliss independence analysis, 1 = strongest, 10 = weakest (Figure 5D). Patient IDs are chosen arbitrarily for reference to Figure 5C.

### The combination of HU and VPA significantly inhibits disease progression in human xenograft models of AML

To evaluate the capacity of combination therapy *in vivo*, the compounds were evaluated in both an OCI-AML3-derived orthotopic model of AML and a primary patient-derived AML xenograft (PDX) (Figure 6). In both models AML cells were engrafted into NOD/SCID IL2rγ^null^ (NSG) mice. The dosing regime for monotherapies and combination treatment was identical in both experiments, though the treatment initiation date differed to reflect differing disease burdens. Preliminary toxicity studies indicated the dosing regime to be well tolerated with minimal adverse effects (Supplementary Figure 2A). The survival curve of the OCI-AML3 orthotropic model illustrates clearly that single agent treatments provoked limited disease response preclinically. Remarkably, a significant increase in survival was observed in combination treated animals compared with controls (*p* = 0.0003), VPA (*p* = 0.0001) and HU (*p* = 0.0014) treated animals (Figure 6A). In a second *in vivo* experiment using an aggressive primary AML PDX model, optical imaging with fluorescently conjugated, multiplexed monoclonal antibodies targeting human leukaemia cells was employed [31, 32]. The purpose of this study was to visualise and quantify the efficacy of the combination *in vivo*. To facilitate imaging therapy was initiated in the stages of advanced disease [31]. *In vitro* studies performed on the primary AML cells used to generate the PDX model confirmed the superiority of the combination compared with monotherapies (Figure 6B). Disease progression before (Day 21) and after (Day 28) treatment was monitored using optical imaging providing a powerful insight into the therapeutic impact of the treatments employed. Combination therapy significantly reduced the total fluorescence of pooled animals at day 28 compared to both monotherapies (vs. HU *p* = 0.0005, vs. VPA *p* = 0.0002) and controls (*p* = < 0.0001), (Figure 5D and 5E). Combination of HU and VPA at the doses and schedules employed did not adversely affect the body weights or condition in either study (data not shown). Furthermore, despite being treated during advanced stage disease, mice treated with the combination typically survived longer compared with monotherapies and control animals, though this observation was not statistically significant (Supplementary Figure 3A). These data suggest that the combination of HU and VPA can impact upon aggressive AML models of late stage palliative AML therapy. Finally, Figure 6F provides a schematic overview of the mechanism described in the article illustrating the molecular insults attributed to HU and VPA, and how the wt expression of p53 ultimately determines cell death or survival in AML cells.

## DISCUSSION

Enhanced efficacy with combination therapy was observed to varying degrees in four AML cell lines (MV4– 11, OCI-AML3, MOLM-13, HL-60) compared with single agent treatment (Figure 1). At clinically relevant doses the mutational status of the p53 protein significantly influenced combination efficacy (Figure 1E–1G). The primary AML cells examined were predominantly p53 wild-type and therefore should reflect the general AML patient population (Table 1). The tendency of enhanced combination effect was recapitulated in these samples (Figure 5) but the relatively limited efficacy observed in some wild-type p53 patient cells (I and H) suggests that other factors may also influence therapy response. When comparing MOLM-13 shp53 and MOLM-13 wt p53 cells combination efficacy proved to be significantly more efficient in those cells containing wt p53. Whilst approximately 90% of AML patients are deemed p53 wild-type, a recent study suggests that some of these patients may harbour residual p53 mutant clones that are infrequent and typically undetected [28]. This suggests that in the context of chemotherapeutic regimes, low abundance mutated p53 clones may be selected for expansion. To examine whether the HU and VPA share this therapeutic limitation we performed a treatment recovery assay in MOLM-13 shp53, MOLM-13 wt p53, HL-60 and OCI-AML3 cells (Figure 2). Importantly, wt p53 was revealed to be critical for mitigating cell cycle recovery following combination induced arrest.

It is recognized that HU specifically targets cells during DNA synthesis by depleting the available nucleotide pool [14, 33]. Following 24 hrs of combination treatment cell cycle analysis showed that OCI-AML3 cells were predominantly found in S-phase and therefore vulnerable from potential genotoxicity incited by HU (Figure 3A). In addition we assessed p21 expression during drug exposure. It is reported previously that HU induced down-regulation of p21 may be required for combination driven cell death. Our results confirmed the reduced p21 expression in the presence of HU and illustrated that VPA substantially slows passage through S-phase in combination treated cells. By altering the sequence of drug exposure (delaying addition of HU by 24 hours) cells were prevented from entry to S-phase and protected from apoptosis. Together these observations highlighted the importance of the cooperative cell cycle arrest previously attributed to these compounds [23]. They also suggest that when operating synergistically, the compounds depended predominantly on HU driven DNA damage to provoke cell death.

The effects of VPA on leukemic cells are diverse and pleiotropic [34]. The capacity to influence various cellular processes whilst maintaining low toxicity may partly explain its amenity to combination strategies. Previous studies have implicated HDAC inhibitors in the dysregulation of homologous recombination repair (HRR) [35, 36]. Our study confirms this property is operative in VPA treated AML cells. In combination treatment, where cells had incurred substantial DNA DSBs, Rad51 staining appeared reduced, diffuse and absent of the characteristic nuclear foci. Rad51 nuclear foci are associated with the proteins functional role in the repair of DSBs and their presence is considered a valid estimation of HRR capacity [37, 38]. Interestingly, increased expression of Rad51 has been suggested as a mechanism of chemo-resistance in FLT3 mutated AML [37]. HU induced DSBs increase corresponding to dose and drug incubation time [39]. At low concentrations or reduced exposure times, cellular capacity to recover from stalled replication forks is sufficient to avoid incitement of DSBs. This is conveyed by the absence of γH2AX expression flowing 24 hrs treatment with HU, but the subsequent increase at 48 hrs (Figure 4A–4B). However, the dramatic increase of combination-induced γH2AX compared to control and mono-treatments exemplifies the capacity for VPA to enhance the potency of HU.

*In vivo* efficacy of the drug combination was assessed in two aggressive mouse models of AML (Figure 6) [31, 32]. These studies provided key preclinical indication that repurposing of these compounds as a low-toxic combination therapy may have clinical value in AML. Our second model assessed combination therapy in a patient derived xenograft (PDX) model of AML. Multiplexing of fluorescently conjugated monoclonal antibodies enabled precise imaging of disease progression before and after therapy. We observed no adverse effects in either model upon treatment with the combination of VPA and HU (Supplementary Figure 2A). Treatment of the PDX animals occurred during advanced disease to facilitate imaging thus potentially inhibiting therapeutic efficacy in regards to survival (Supplementary Figure 3A). Nevertheless, *in vivo* imaging provided a clear indication that both the leukemic burden and dissemination of AML blasts was significantly reduced in mice receiving combination therapy when compared to control and mono-therapy groups.

We observed difference in efficiency of HU and VPA in cell line models and a varying effect in the primary AML cells tested *in vitro*, underscoring the future possibility to identify responders or non-responders of HU and VPA based on molecular and biological characteristics of the AML disease. Several case reports [21] together with our animal models have suggested the compounds are unlikely to result in toxicity when administered in combination, indicating the feasibility to test HU and VPA in a controlled clinical trial of unfit elderly AML patients. We believe that repositioning of HU and VPA as a combination therapy could significantly enhance the palliative care of patients unsuited to intensive chemotherapy, particularly if non-responders can be predicted prior to initiation of treatment

## MATERIALS AND METHODS

### Cell lines, primary AML cells and healthy PBMCs

Seven leukemic cell lines were included in the study, OCI-AML3 (DSMZ), MV4–11, HL-60, KG1-A, THP-1, K562 and MOLM-13 (ATCC). p53 knocked down MOLM-13 cells and empty vector (MOLM-13 shp53) were generated by retroviral transfection for stable expression of shRNA against p53 using the pRETRO SUPER-p53 vector [5]. The concentration of puromycin was steadily increased to 400 μg/ml over a two-week period. Primary AML cells were acquired from patients at Haukeland University Hospital following informed consent. Approval was obtained from the regional Ethics Committee (REK Vest; http://helseforskning.etikkom.no; Norwegian Ministry of Education and Research). Normal peripheral blood lymphocytes were obtained from healthy blood donors (Blood bank, Haukeland University Hospital, Bergen, Norway).

### Cell death assays

Evaluation of cell death and apoptosis in cell lines and primary AML cells after drug treatment was performed using Hoechst 33342 staining of nuclear morphology and AnnexinV-propidium idodide (Annexin- PI) detection of apoptosis by flow cytometric analysis. Assays were performed as previously described [5].

### Western blotting

Western Blotting was performed as previously described [5]. The following antibodies were used; Actin (sc- 4778) Chk1 (DCS-310) p53 (Bp53–12), Rad51 (sc-8349) (all from Santa Cruz Biotech), p21 (EA10, Abcam,), γH2AX (DR1017) and H2AX (DR1016) (both from CalBiochem, Germany). Goat-POD- anti-mouse or anti-rabbit antibody secondary antibodies (Jackson ImmunoResearch) were used as appropriate for primary antibody detection.

### Immunofluorescence

Following treatment incubations cells were cytospun onto coverslips followed by fixation and permeabilization with 4% paraformaldehyde for 20 min and ice-cold 99% methanol for at least 20 min at −20 degrees or stored, respectively. Cells were then blocked with 0.5% Bovine Serum Albumin (BSA) (Roche Diagnostics GmbH) in 1XPBS for 15 min before incubation with primary antibody, rabbit Rad51 (1:200, sc-8349), γH2AX (DR1017, CalBiochem, Germany) or Nsb-1 (D6J5I, Cell Signalling Technology) diluted in 1XPBS with 0.5% BSA at 4°C over night. After three washes in 1XPBS, cells were incubated with secondary antibody (1:5,000 of Alexa 568 goat anti-rabbit (Invitrogen Molecular Probes)) diluted in 1XPBS with 0.5% BSA, was performed in the dark for 1 hour at room temperature. Finally, the coverslip was washed three times with 1XPBS, dipped once in water and mounted in 5 μl Fluoro-gel II with DAPI (Electron Microscopy Sciences, PA, USA). Images of were acquired with a Zeiss Axio Observer Z1 inverted microscope (Carl Zeiss Microimaging GmbH, Germany) and analyzed by the AxioVision 4.8.2 software.

### Cell cycle analysis

Cells were fixed in 70% ethanol in PBS overnight. For DNA content analysis cells were pelleted and resuspended in PBS containing 1 mg/ml RNase (Sigma Aldrich) and 10 mg/ml PI, incubated at room temperature for 30 min, then analysed using the Accuri C6 flow cytometer (BD Sciences).

### H2AX and γH2AX expression assay

The assessment of H2AX expression in OCI-AML3 cells was performed using the H2AX Muse™ H2A.X Activation Dual Detection Kit (Millipore). 1 × 10^5^ cells were analyzed from each sample condition following 48 hrs drug exposure. Samples were prepared in strict accordance to the manufacturer's procedure and run on the Muse^®^ Cell Analyzer flow cytometer.

### Monoclonal antibody conjugation

Monoclonal antibodies (mAbs) CD45 (clone F10–89–4), CD33 (clone WM53), HLA ABC (clone W6/32; all AbD Serotec) were conjugated to Alexa Fluor 680 using the SAIVI Alexa Fluor 680 Labelling Kit (Invitrogen) as described [32]. Protein concentrations of the Alexa Fluor 680–conjugated mAbs degree of labelling were determined using a Nanodrop 1000 spectrophotometer (Thermo Fischer Scientific).

### Optical imaging

Prior to imaging, mice were depilated and anesthetized with 1–2% isoflurane (Isoba; Schering-Plough), 0.2 L/min of O_2_, and 0.2 L/min of N_2_. NIR images were obtained with the eXplore Optix or Optix MX3 Small Animal Molecular Imager system (ART Inc). NIR imaging scans (λ_ex_ = 670 nm, λ_em_ = 700 LP, laser repetition rate 80 MHz, raster scan points 1 mm apart) were obtained 24 hours after administration of Alexa Fluor 680–labelled mAbs (total mAb concentration of 1 μg/g). Optiview software (Versions 1.04 and 2.02; ART Inc) was use to analyse images and perform fluorescence lifetime gating.

### Mouse xenograft models

All animal experiments were approved by the Norwegian Animal Research Authority and performed in accordance with The European Convention for the Protection of Vertebrates Used for Scientific Purposes. Intravenous injection of OCI-AML3 cells (1.5 × 10^6^ cells/100 μL/mouse) and primary AML cells (5 × 10^6^ cells/100 μL/mouse) was performed on female NOD/SCID IL2rγ^null^ (NSG) mice (Vivarium, University of Bergen; originally a generous gift of Dr Leonard D. Shultz, The Jackson Laboratory).

### Dosing regime

VPA (100 mg/mL, Orfiril) was sterile filtered from the vial. HU (76.05 g/mol, Sigma

Aldrich) was daily prepared with saline to a concentration of 200 mg/mL.

In both xenograft experiments mice were treated for five consecutive days. In the primary AML model therapy was initiated day 21, whilst in the OCI-AML3 models treatment began day 14. Animals were divided into four groups, control animals receiving 50 μl sterile saline, VPA 350 mg/kg/day, HU 500 mg/kg/day, and combination (VPA 350 mg/kg/day + HU 500 mg/kg/day). All groups contained a minimum of 5 animals and all dosing was performed by intraperitoneal injection.

### Statistical analysis

In cell death assays results are displayed as the mean +/− standard deviation. Synergism was calculated by the Chou-Talalay method [40] or by Bliss Independence analysis [41]. In both *in vitro* and *in vivo* studies statistical significance between the averages of varying treatment groups were determined using a two-tailed Student *t* test. *In vivo* survival data was evaluated using the Kaplan and Meier analysis method. A one-way analysis of variance (ANOVA) was performed to ensure no statistical significant difference in weights between the animals in the treatment groups.

## SUPPLEMENTARY MATERIALS FIGURES


