# Supplementary material for: Hydroxyurea synergizes with valproic acid in wild-type p53 acute myeloid leukaemia
Source: Oncotarget. 2016 Jan 23;7(7):8105–18. doi: 10.18632/oncotarget.6991 (PMC4884979; doi:10.18632/oncotarget.6991)
Supplement: Supplementary file 1 [file oncotarget-07-8105-s001.pdf]

Hydroxyurea synergizes with valproic acid in wild-type p53 acute myeloid leukaemia

Supplementary Materials

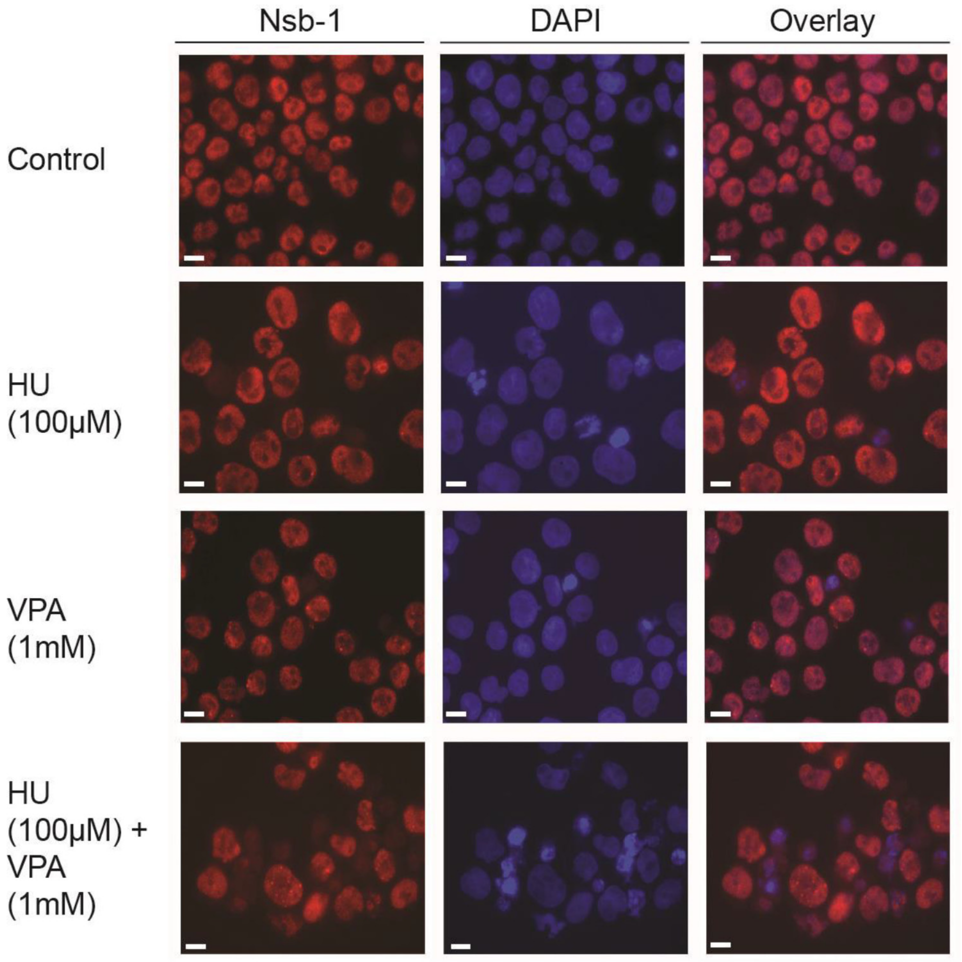

Supplementary Figure S1: (**A**) OCI-AML3 cells were treated for 48 hrs with HU (100 μM), VPA (1 mM) or in combination, before cytospun, fixed and immunostained for Nsb-1. Representative image of three independent experiments is shown (*N* = 2).

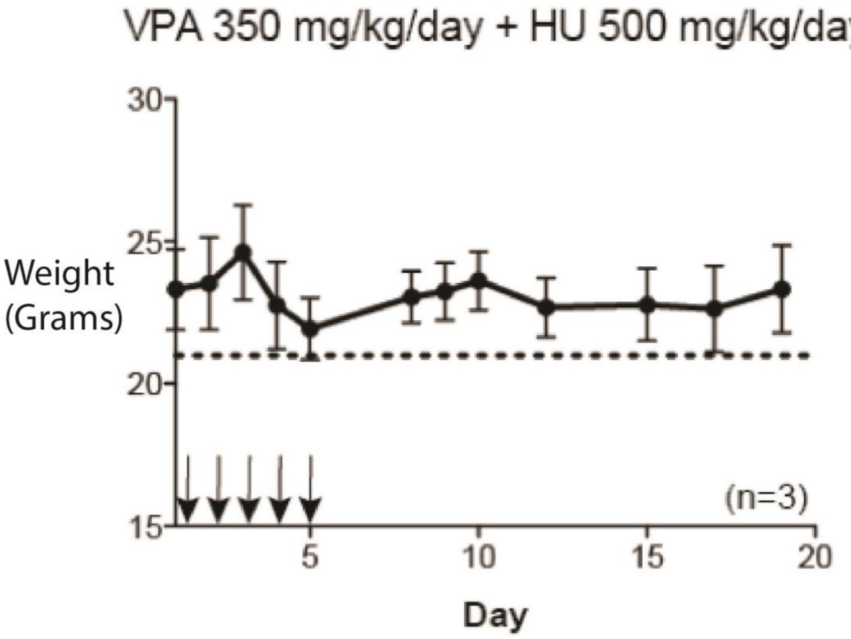

Supplementary Figure S2: (**A**) Preliminary animal toxicity data. Weight graphs of female NOD/SCID IL2rγ^{null} (NSG) mice undergoing the 5 day combiation treatment regime administered in leukeamia mouse models. Arrows indiate dosing days. Dotted line represents 10% weight loss and would be considered toxic.

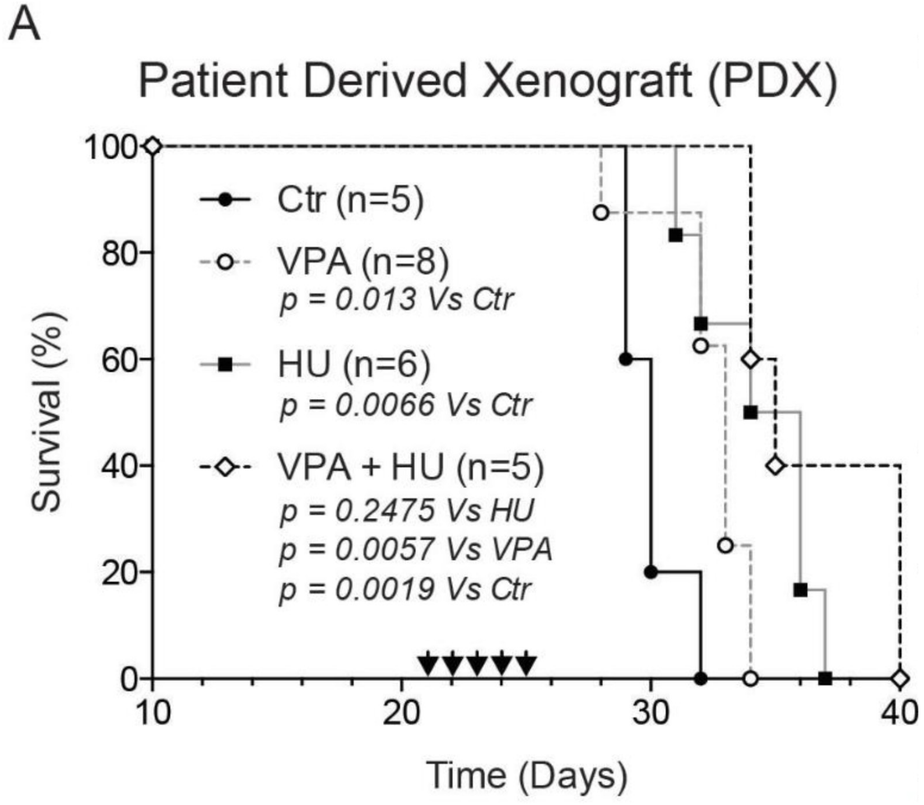

Supplementary Figure S3: (**A**) Survival data presented in Kaplan-Meyer curve illustrating the efficacy of HU and VPA and increased survival of combination therapy for the primary PDX model of AML (log-rank *P* = 0.0019 vs controls, *P* = 0.2475 vs HU, *P* = 0.0057 vs VPA). Arrows indicate days on which animals were dosed with both compounds.
